# Role of Intramuscular Connective Tissue in Water Holding Capacity of Porcine Muscles

**DOI:** 10.3390/foods11233835

**Published:** 2022-11-28

**Authors:** Jingfan Wang, Ping Yang, Dong Han, Feng Huang, Xia Li, Yu Song, Hang Wang, Jiqian Liu, Jian Zheng, Chunhui Zhang

**Affiliations:** 1 Key Laboratory of Agro-Products Processing, Ministry of Agriculture and Rural Affairs, Institute of Food Science and Technology, Chinese Academy of Agricultural Sciences, Beijing 100193, China; 2 Laboratory of Chemistry of Natural Molecules, Gembloux Agro-Bio Tech, University of Liege, 5030 Gembloux, Belgium; 3 Inner Mongolia Xibei Restaurant Group Co., Ltd., Beijing 100161, China

**Keywords:** pork, water holding capacity, intramuscular connective tissue, muscle fiber, protein denaturation

## Abstract

Background: This study evaluated the influence of intramuscular connective tissue (IMCT) on structural shrinkage and water loss during cooking. Longissimus thoracis (LT), semimembranosus (SM) and semitendinosus (ST) muscles were cut and boiled for 30 min in boiling water, followed by detection of water holding capacity (WHC), tenderness, fiber volume shrinkage and protein denaturation. Results: Compared with LT and SM, ST had the best WHC and lowest WBSF and area shrinkage ratio. The mobility of immobilized water (T22) was key to holding the water of meat. ST contained the highest content of total and heat-soluble collagen. On the contrary, ST showed the lowest content of cross-links and decorin, which indicate the IMCT strength of ST is weaker than the other two. The heat-soluble collagen is positively correlated to T22. Conclusions: The shrinkage of heat-insoluble IMCT on WHC and WBSF may partly depend on the structural strength changes of IMCT components rather than solely caused by quantitative changes of IMCT.

## 1. Introduction

Meat processing has been of great concern, and cooking is a common heat treatment applied to meat. Cooking contributes to causing structural and chemical changes in the meat; with cooking, meat becomes more palatable [1]. However, cooking affects muscle quality, such as weight loss, juiciness, and area shrinkage. One of the most important factors is the water-holding capacity (WHC), which is a critical indicator that affects weight loss, area shrinkage, and juiciness during the cooking of meat [2]. There are two main factors, including protein denaturation and myofibrillar structural shrinkage, which can influence the WHC of cooked meat [3]. At different heating temperatures, muscle tissue shrinkage results from different protein denaturation. The shrinkage in the sarcomere structure decreases the WHC of muscles. During heat-induced shortening, the myofiber structure can be torn by the force that is created by the contraction of cross-bridges consisting of myosin and actin filaments [4]. 

Water in the myofibrillar structure is expelled by the structural change caused by thermal denaturation [5]. The shrinkages of muscle structure during cooking can be summarized as follows: the transversal shrinkage occurs mostly at 40–60 °C, which results from myofibrillar protein denaturation. Transversal shrinkage enlarges the gap between the myofibers and the surrounding endomysium, causing the majority of water loss. The longitudinal shrinkages to the fiber axis take place mainly at 60–70 °C; the intramuscular connective tissue (IMCT) network and myofibers shrink collaboratively [6]. Different proteins have different thermal denaturation temperatures, such as myosin, actin and collagen. The meat quality traits differ in diverse muscles in terms of myofiber characteristics [7,8].

There has always been some dispute about the value of IMCT in meat heating [9]. The force generated by the contraction of IMCT drives fluid out of myofibres [6,10]. Connective tissue is made up of cells and extracellular matrix. There is a matrix of proteoglycans embedded in collagen fibers [11]. The mature intramolecular cross-links are essential for the in vivo mechanical functions of collagen [9]. After the synthesis of collagen fibres by fibroblasts, cross-links are formed immediately [9]. The temperature of thermal denaturation of IMCT collagen is usually between 58–67 °C. It is generally accepted that, above 65 °C, collagen contraction leads to meat shrinkage, which generates a greater cooking loss [9,12]. However, there still needs more convincing research to support this point. The investigative results of the possible mechanisms are different from the possible way that cooking loss is correlated to collagen shrinkage. A study investigating [11] beef muscles found that cooking loss was not correlated to collagen content [13]. The improvement of cooking on IMCT, as well as their influence on meat qualities, remains to be investigated.

There is conjecture that thermally insoluble collagen is currently more acceptable [14]. The hypothesis for the present study was that the heat-insoluble IMCT fraction would influence IMCT network shrinkage and may contribute to squeezing fluid out of the matrix. Heat soluble IMCT fraction would influence water distribution, especially the immobilized water. The heat solubility affects WHC. Consequently, by comparing WHC and shrinkage differences between three distinct porcine muscles, the objectives of the research were (1) to investigate the influence of IMCT on structural shrinkage and water loss during cooking, and (2) to explore the change of quantity and quality of IMCT during heating.

## 2. Materials and Methods

### 2.1. Samples Collection

Five pork carcasses (Duroc × Landrace × Yorkshire crossbred, 6 months of age, 110 ± 10 kg) were purchased from a local market. Three different pork muscles were obtained: *longissimus thoracis* (LT, n = 11), *semimembranosus* (SM, n = 11) and *semitendinosus* (ST, n = 11). There were three batches. For the first batch, three samples of each muscle (LT, SM and ST) were collected from one pork carcass of crossbreed animals. For the other two batches, four samples of each muscle (LT, SM and ST) were collected from two pork carcasses. All the samples were split after precooling at 4 ± 1 °C for 24 h. Each meat steak was 100 g (4 × 4 × 5 cm). The samples were sealed in a low-density polyethylene bag individually. After packaging, samples were heated in a circulating thermostatic water bath (TESTO735, TESTO, Schwarzwald, Germany) at 100 °C for 30 min to (the core temperature reached about 97 °C). After cooking, samples were quickly cooled in the ice water.

### 2.2. Warner-Bratzler Shear Force (WBSF)

The method to determine Warner-Bratzler shear force (WBSF) was described by Holman et al. [15] and Qian et al. [16]. All blocks were approximately 50 mm × 10 mm × 10 mm high, and parallel to muscle fibers. Measurements were carried out on a tenderness testing machine (C-ML3B, TENOVO, Beijing, China). Ultimately, the result of WBSF was presented as a unit of force.

### 2.3. Water Holding Capacity

#### 2.3.1. Cooking Loss

According to the method of Jeong et al. [17], samples were wiped dry and weighed. The cooking loss (%) was calculated according to:

(1) $$\mathrm{Cooking}\;\mathrm{loss}\;\left(\%\right)=\frac{m_{a}-m_{b}}{m_{a}}\times 100\%$$

where $\left(m_{a}\right)\;\mathrm{and}\;\left(m_{b}\right)$ represent the weight of raw and cooked samples.

#### 2.3.2. Low-Field Nuclear Magnetic Resonance (LF-NMR) Measurements

Water distribution was measured according to the method of Li et al. [18]. The transverse relaxation, *T*_2_, were tested on a *LF-NMR* Analyser (PQ-001, Niumag Electric Corporation, Shanghai, China; magnetic field strength: 0.5 Tesla; probe coil diameter: 60 mm; operating temperature: 27 °C; spectrometer frequency: 23 MHz). *T*_2_ was determined using the Carr–Purcell–Meiboom–Gill sequence (CPMG) with phase cycling to remove the artifacts. Data were expressed by using the MultiExp Inv Analysis software (Niumag Electric Corporation, Shanghai, China). Three relaxation *T*_2_ water populations and proportions (bound water, immobilized water and free water) were obtained [19]. The nuclear magnetic resonance spin–spin relaxation (*T*_2_) was measured by *LF-NMR*. Three populations can be classified as bound water (*T*_21_, 0.1–10 ms), immobilized water (*T*_22_, around 100 ms) and free water (*T*_23_, 100–1000 ms) [16,20,21]. The relative percentages of the three fractions of water are showed as *A*_21_, *A*_22_ and *A*_23_.

### 2.4. Structural Shrinkage

#### 2.4.1. Area Shrinkage

According to the method of Kong et al. [22], the area shrinkage ratio was determined as the percent reduction between raw and cooked samples. Transverse and longitudinal shrinkages were calculated as the area difference of raw and cooked samples along and across the muscle fibers, respectively.

(2) $$\mathrm{Transverse}\;\mathrm{shrinkage}\;\left(\%\right)=\frac{x_{1}-x_{2}}{x_{1}}\times 100\%$$

(3) $$\mathrm{Longitudinal}\;\mathrm{shrinkage}\;\left(\%\right)=\frac{y_{1}-y_{2}}{y_{1}}\times 100\%$$

where $x_{1}$ and $x_{2}$ represent the area of raw and cooked samples across the muscle fibers, respectively; whereas, $y_{1}$ and $y_{2}$ represent the area of raw and cooked samples along the muscle fibers, respectively.

#### 2.4.2. Microstructure Measurements

##### Histological Analysis

The 4 μm thickness transverse sections were cut from the 1 × 1 × 2 cm meat strips. The sections were stained by the Picrosirius method of Li et al. [23]. Microphotographs were obtained by an Eclipse microscope (Nikon CI-S, Nikon, Tokyo, Japan) fitted with a CCD imaging system (Michrome 5 PRO, SONY, Fuzhou, China).

##### Scanning Electron Microscopy (SEM)

According to Krystyna et al. [24], the microstructural changes across the muscle fibers were evaluated by a scanning electron microscope (SEM). The 3 × 3 × 5 mm strips, cut along the direction of muscle fibers, were fixed with 3% glutaraldehyde for 48 h. Samples were rinsed with distilled water for 1 h. An ethanol series was used to dehydrate samples. After drying, samples were sputter-coated with gold (Eiko IB-5, Hitachi, Tokyo, Japan). Cross-sections of myofibers were detected with a scanning electron microscope (Quanta 200 FEG, FEI, Eindhoven, Netherlands); the magnification was ×300.

##### Transmission Electron Microscopy (TEM)

The ultrastructural changes along the direction of myofibers were observed using a transmission electron microscope (TEM) by Wang et al. [25]. Muscle strips were fixed with 3% glutaraldehyde for 48 h, post-fixed with 1% osmium tetroxide for 2 h, and then washed with 0.1 M PBS. Afterwards, samples were dehydrated by gradient ethanol series. Sections were prepared on Leica UC6 ultra-microtome, stained with uranyl acetate and lead citrate, and detected under a transmitting electron microscope (H-7500, Hitachi, Tokyo, Japan). The magnification was ×30,000. Image J software was used to measure sarcomere length, which was calculated as the distance between two Z lines.

### 2.5. Surface Hydrophobicity

According to Mitra et al. [26], the surface hydrophobicity of samples was evaluated by binding of hydrophobic chromophore bromophenol blue (BPB); 2 g of samples were mixed in 20 milliliters of PBS (20 mM, pH 6.0). PBS was used to adjust the total protein concentration to 5.0 mg/mL. 1.0 mL of suspension was added to 200 μL of BPB (1.0 M) and vortexed. A control was prepared by adding 200 μL of 1.0 M BPB to 1.0 mL of PBS (20 mM). Afterwards, the samples were centrifuged (15 min 2000× *g*). The supernatant was removed and then diluted 10 times with PBS. The absorbance was evaluated at 595 nm. The amount of bound BPB was calculated by the equation:

(4) $$\mathrm{Bound}\;\mathrm{BPB}\;\left(\mu g\right)=\frac{OD_{Control}-OD_{Sample}}{OD_{Control}}\times 200\;\mu g$$

### 2.6. IMCT Traits

#### 2.6.1. Collagen Content

The determination of total and soluble collagen was measured according to Starkey et al. [27] with slight modifications. Total collagen content was conducted in triplicate from 0.1 g of freeze-dried muscle powder. Samples were added to 3 milliliters of H_2_SO_4_ (3.5 mM) and then hydrolyzed at 106 °C for 16 h. After hydrolysis, the total volume was adjusted to 50 mL, which was subsequently filtered; 0.25 mL of NaOH (1.5 M) and 3.75 mL of water were added to 1 mL of the filtrate. The hydroxyproline content was evaluated at 558 nm. Total collagen content was calculated by the formula:

(5) $$\mathrm{Total}\;\mathrm{collagen}\;=\frac{\mathrm{Hydroxyproline}\times 7.25\times \mathrm{Weight}}{1000\times 250}$$

Heat-soluble collagen content was determined by suspending 1.5 g of freeze-dried muscle powder in 10 milliliters of water. The samples were heated at 80 °C for 2 h and vortexed every 0.5 h. The samples were centrifuged (30 min 1500× *g*) and then filtered. A half milliliter of the filtrate was taken and combined with 3 milliliters of H_2_SO_4_ (3.5 M). This solution was subsequently heated for 16 h at 106 °C. Water was added to the hydrolysate to make a 10 mL volume. One milliliter of the diluted filtrate was used for measuring the hydroxyproline content. The heat-soluble collagen content was calculated by the formula:

(6) $$\mathrm{Heat}-\mathrm{soluble}\;\mathrm{collagen}\;=\frac{\mathrm{Hydroxyproline}\times 7.25\times \mathrm{Weight}}{1000\times 400}\;$$

#### 2.6.2. Cross-Links and Decorin

Cross-links were determined as described by Wang et al. [28]; 250 mg of freeze-dried muscle samples were acid hydrolyzed with 4 milliters of HCl (6 M) at 110 °C for 12 h, and then centrifuged (5 min 16,000× *g*). 1 mL of NaOH (6 M) and 1 mL of Tris (1 M) were mixed to 1.0 mL of the acidic supernatant. The final pH was adjusted to 7.0–7.2. Analysis for decorin, 500 mg of freeze-dried muscle samples were mixed in 10 milliliters of PBS. The suspension was subjected to 2 freeze–thaw cycles. Afterwards, samples were centrifuged (15 min 1500× *g*). The supernatant was collected for further analysis. The content of pyridinoline cross-links and decorin were measured using the enzyme-linked immunoassay (ELISA) kit (Shanghai Jianglai Biotech, Shanghai, China) following the manufacturer’s instructions.

### 2.7. Statistical Analysis

Data were statistically analyzed by general linear model procedures of SPSS Statistics 26.0. Porcine muscle was regulated as a fixed factor and three batches as a random factor. Duncan’s test was performed to identify significant differences at *p* < 0.05.

## 3. Results and Discussion

### 3.1. Water Holding Capacity and WBSF

**Figure 1:**
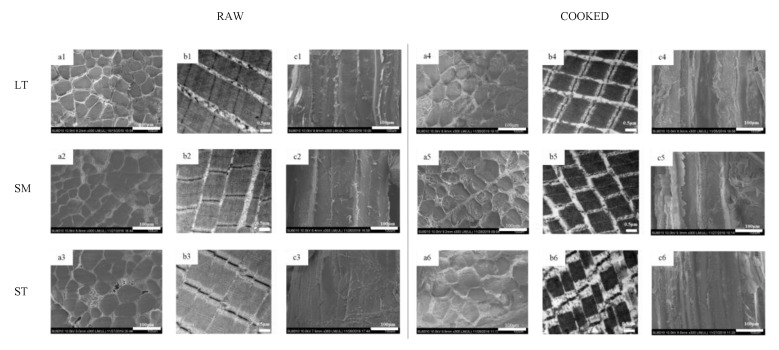
Microstructure and ultrastructure observation of porcine *longissimus thoracis*, *semimembranosus* and *semitendinosus*. (**a**) transversal SEM micrographs; (**b**) TEM micrographs; (**c**) longitudinal SEM micrographs; 1, LT; 2, SM; 3, ST.

WBSF was highly correlated with tenderness. As shown in Table 1, the value of WBSF was highest (*p* < 0.05) in LT muscle. The contraction and fracture of myofibers may contribute to the shear force [16]. The higher WBSF in LT was correlated to its microstructural shrinkage, which is shown in Figure 1. LT showed the biggest WBSF might be correlated to cross-links. In accordance with Wang et al. [28], WBSF was positively correlated with the intrinsic IMCT traits (the content of mature cross-links and decorin). In addition, the lower shear force in ST may result from the increase in collagen solubility, which is shown later.

During heating, the water content of meat decreases due to its shrinkage. The results of the cooking loss of three distinct muscles is displayed in Table 1. Compared to ST, the cooking loss of LT and SM was significantly higher (*p* < 0.05). Water loss within the myofiber would increase, for changes in the myofibrillar structure and denaturation of protein were affected by the improvement of temperature and time [29]. The reasons for this observation might be correlated to the differences in ultrastructure among the three porcine muscles.

Three populations can be divided into bound water (*T*_21_, 0.1–10 ms), immobilized water (*T*_22_, around 100 ms) and free water (*T*_23_, 100–1000 ms) [16,20,21]. Their relative percentages are shown as *A*_21_, *A*_22_ and *A*_23_ in Table 1. A longer *T*_2_ relaxation time corresponds to a poorer WHC, which might result from the reduction of water-protein interaction [30]. The result of *LF-NMR* is in agreement with cooking loss. A significant change of the relaxation time of bound water (*T*_21_) was not displayed among LT, SM and ST. This is consistent with Pearce [31], who found that bound water may keep still in response to any microstructural changes (such as freezing and heating) in the meat matrix. Water distribution significantly changed among the three muscles (*p* < 0.05). *T*_22_ and *T*_23_ of ST were significantly longest (*p* < 0.05), and *A*_22_ was significantly longer with ST than LT (*p* < 0.05). The increment of *T*_22_ and *A*_22_ indicated that the mobility of immobilized water was enhanced [25], that is to say, the immobilized water mobility of ST was enhanced. The longer *T*_22_ in the meat matrix is related to the higher mobility of entrapped protons [21]. H-proton has fewer degrees of freedom, which indicates that the hydrogen bond energy of retained immobilized water is higher [32]. The increase of *T*_23_ and *A*_23_ may result from the shifting of immobilized water to free water, which may result from the hydrogen bonds between water and protein [16]. Transformation of *A*_22_ to *A*_23_ could be facilitated by heat-induced sarcomere microstructural shortening (as shown in Figure 1). These shrinkages would squeeze out the intra-myofibrillar water and further result in cooking loss. ST showed prolonged *T*_2_ relaxation times, which suggested that the existence of greater mobile water component in it.

### 3.2. Structural Shrinkage

#### 3.2.1. Volume Shrinkage

The structural muscle contraction, caused by the denaturation of protein, resulted in the major loss of meat mass during cooking [33]. As pork shrinks, the mass loss is made up of water and soluble proteins, as well as some other compounds [34]. Table 2 displays the changes in transverse and longitudinal shrinkage ratios as affected by heating. The area shrinkage ratio showed a similar trend to cooking loss; LT showed the biggest cooking shrinkage (*p* < 0.05). The results of volume shrinkage are consistent with cooking loss. Transversal shrinkage of muscle fibers will account for the majority of the cooking loss. But as the temperature rises, denaturation of collagen and longitudinal shrinkage is responsible for attached loss [35]. Compared with ST, the volume and longitudinal shrinkage of SM did not show a significant change, which may indicate that, for SM, the longitudinal shrinkage plays a leading role during the volume shortening.

#### 3.2.2. Microstructural Shrinkage

The microstructure and ultrastructure of myofiber in three muscles are shown in Figure 1. After cooking, all three muscles exhibited distinct fiber diameter and sarcomere length shrinkage. Gaps between fiber bundles were much more visible after cooking (Figure 1(a1–a6)). The differences in the content and structure of IMCT may be attributed to the results of transversal shrinkage. During heating, water is released from the myofibers, which would be squeezed out by the collagenous network contraction.

The thermal transformation of collagen is gradual and highly reliant on the ability of collagen to shrink [36]. The heating had severe destruction on the muscle structure, especially the Z-lines (Figure 1(b1–b6)). ST showed the most serious disruption of the I-band. It is proposed that the reduction of vulnerable I-region leads to the diminishment of proteolysis of myofibrils in shortened meat [3]. There were granulation and coagulation changes in IMCT (Figure 1(c1–c6)). The epimysium granulation of ST was much more perceptible. The gel results from the thermal denaturation of collagen wrapped around the muscle fibers [37]. The connection of muscle fiber bundles was loose after cooking. LT showed severe body shrinkage might result from its cross-links and decorin content. The presence of thermal-stable bonds means that cross-links are held at these temperatures. It is inferred that the contraction of heat-insoluble IMCT on WHC may depend, to some extent, on the proteolysis and structural strength changes of IMCT components.

**Figure 2:**
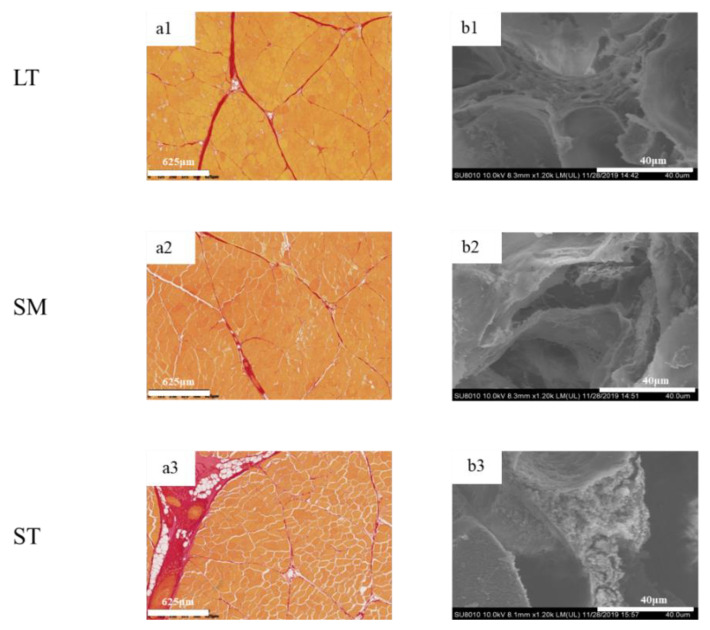
Histological microscopic images and SEM micrographs of porcine *longissimus thoracis*, *semimembranosus* and *semitendinosus*. (**a**) histological microscopic images; (**b**) SEM micrographs; 1, LT; 2, SM; 3, ST.

To determine the differences in IMCT in the three muscles, the digital images are shown in Figure 2. The muscle fibers were stained yellow, while the perimysium were red. LT exhibited myofibers of highly regular diameter and normal fibrillar organization as compared to ST. The amount of intramuscular connective tissue in ST was the highest, while the structural integrity of fiber bundles was compromised. The quantity and quality of collagen are vital in deciding meat tenderness [38].

**Figure 3:**
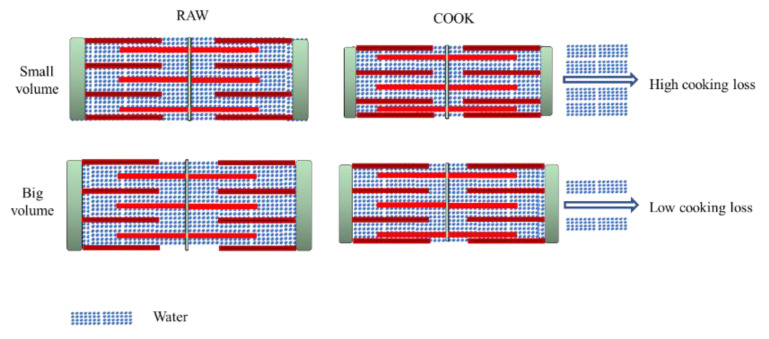
Comparison between cooking loss of different porcine muscles.

The fiber diameter of both samples (raw and cooked) is shown in Table 2. Sarcomere length is a related indicator correlated with water-holding [4]. The water content would increase for the expansion of the muscle fiber lattice spacing [3]. ST displayed the biggest fiber diameter and sarcomere length, no matter if raw or cooked, which indicates ST has much more space to retain water. A large quantity of water exists in the region between thin and thick myofilaments (Figure 3). Due to the protein denaturation, the sarcomere length decreases in a temperature-dependent manner. The decrease in myofiber diameter and sarcomere length alters the distribution of water within the myofibril spaces [39,40]. The Z-disk is closely associated with stable sarcomere against transversal and axial forces transmission along with the myofiber. For ST, the Z-disks of adjacent myofibrils are aligned, which promotes coordinate contraction between individual muscle fibers (Figure 1(b3,b6)) [4]. After cooking, the alignment of Z-disks decreased. The sarcomere length shrinkage of ST is the greatest. The volume of cooked ST is still the biggest, which gives it much more space to hold water. Compared with the shrinkage ratio, the volume has a greater effect on improving WHC.

### 3.3. Protein Surface Hydrophobicity

**Figure 4:**
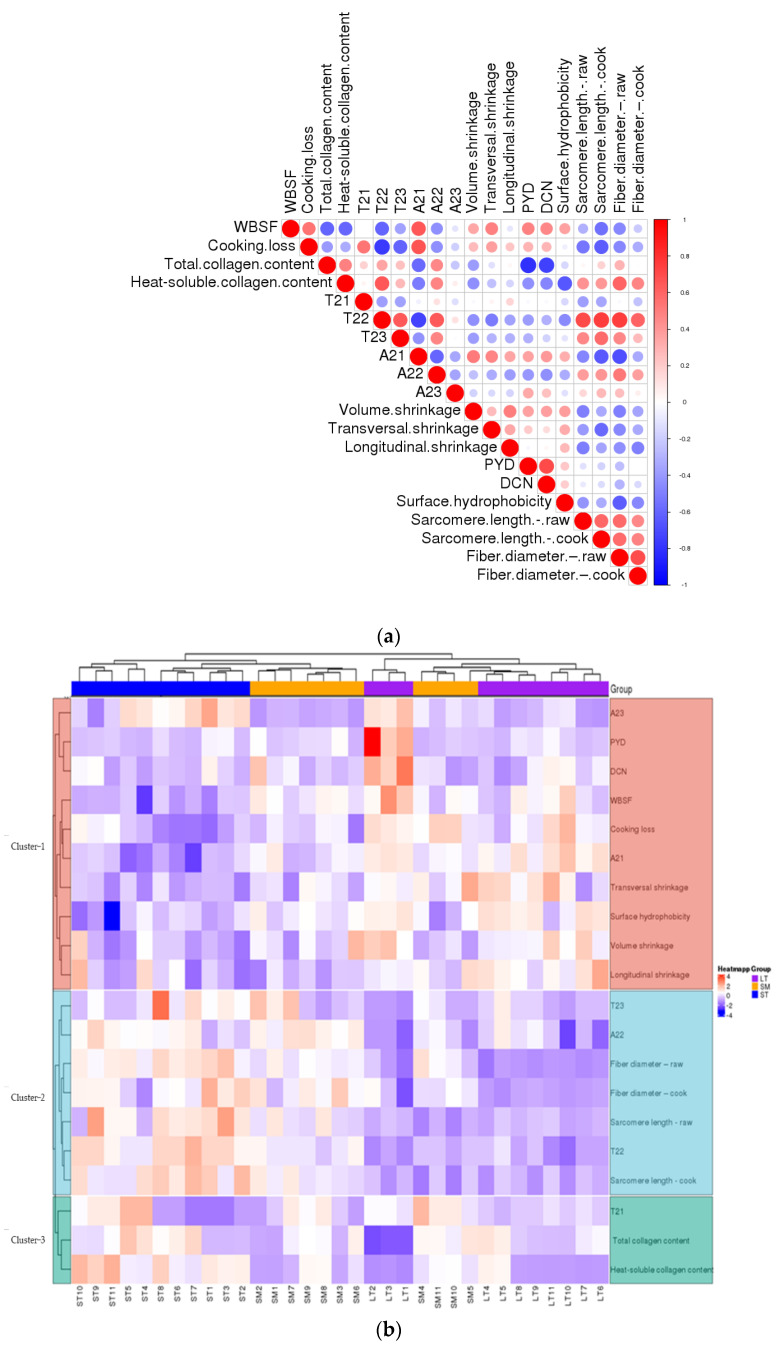
(**a**) The heatmap of correlations between different parameters. (**b**) The heatmap of cluster analysis of porcine muscles and parameters.

Protein surface hydrophobicity could be an appropriate indicator to evaluate protein denaturation, as it monitors sensitive changes in the physical and chemical state of proteins. Differences among the three muscles in the surface hydrophobicity are shown in Table 3. LT showed the highest surface hydrophobicity, which confirmed the greatest break of conformational stability in proteins. The increase of surface hydrophobicity proves the unfolding of proteins and exposure of nonpolar amino acids to the surface [6]. A molecular rearrangement into hydrophobic clusters can promote intra- and intermolecular interactions [41]. LT showed the most serious protein denaturation. It’s interpreted that *T*_22_ was negatively correlated with surface hydrophobicity (Figure 4a). To some extent, surface hydrophobicity was negatively correlated to the water-holding capacity of porcine muscles. A shorter *T*_22_ indicated lower mobility of entrapped protons and therefore reflected more protein denaturation. These results were accordant to Table 1 and Song [21].

### 3.4. IMCT

The content of total collagen and heat-soluble collagen are shown in Table 3. Compared to LT, total collagen in ST was higher (*p* < 0.05). During heating, the increase in the total collagen content might result from the increase in cooking loss and the transformation of collagen to gelatin [37]. The difference between heat-soluble collagen was statistically significant (*p* < 0.05) among the three muscles, while the content of cross-links and decorin were the lowest. The results of cross-links and decorin suggested that the stability of collagen fibers of LT was better than ST. The results are accordant to Lepetit [36]. It’s supposed that the higher the amounts of cross-links, the higher the thermal contraction of collagen fibers [36]. Furthermore, the development of cross-links in muscle tissue might be mediated by decorin [42]. Both SM and ST muscles are attached to the aitchbone and myofibers are stretched longitudinally over the knee joint. The ST and SM muscles are connected directly to the bone and are exercised frequently, therefore, their amount and heat solubility of IMCT are higher than LT. These results are consistent with the hypothesis proposed by Purslow [14], who reported that heat-insoluble collagen occurs in a weak pool and a strong pool. The weak pool was less stable, more easily denatured and solubilized during heating. The strong pool was more cross-linked, and resistant to heating and proteolysis. When heated above 60 °C, the strength of the cooked material is determined by the strong pool.

Heat-soluble collagen content is negatively correlated with WBSF. A proportion of the heat-insoluble collagen decreased during heating, and another part of collagen is efficiently heat-insoluble. The increase of heat-insoluble cross-links was positively related to increase WBSF. Accordingly, both the quantity and quality of collagen conduce to tenderness. A negative correlation was observed between the cross-links, decorin and the heat-soluble collagen (Figure 4a). The results are consistent with the conclusion that the divalent cross-links were replaced by mature cross-links, which were associated with reduced thermal solubility of collagen [9]. Heat solubility might result from different skeletal muscle that has different types of collagen. Previous studies have shown that type I collagen is more readily resolved by heat than type III [43,44]. The reduction in cross-links indicated the decline of cross-links to bind three polypeptide subunits [28,45,46], which induced a decrease in the intensity of IMCT. Decorin acts as a spacer in the lateral assembly of the collagen molecular structure [28]. It was an indispensable part of collagen to maintain normal tissue function and mechanical properties [28]. The decorin was important in developing the elasticity and strength of collagen fibers [47]. The collagen fiber strength of LT is the greatest among the three porcine muscles. Both the quantity and quality of collagen were key in deciding meat structural changes.

### 3.5. Contribution of Denaturation of Proteins to the Physical Characteristics

The correlations of the porcine muscles were analyzed and presented as a hierarchical clustering heat map (Figure 4). It was a negative correlation between *T*_22_, *T*_23_, *A*_22_, fiber diameter, sarcomere length and cooking loss. *T*_22_ was negatively correlated to volume shrinkage (Figure 4a). The results suggest the structural changes induced by the cooking procedure influence the WHC. The increasement of content (*A*_22_) and mobility (*T*_22_) of immobilized water were key to holding water in the meat. The cluster analysis results between three porcine muscles and physico–chemical characters were analyzed and are shown as a hierarchical heat map (Figure 4b). The variation from different samples within the same muscle type almost presented a similar color, and different muscle types presented different colors, especially LT. The heat-induced shrinkage squeezes out water in the meat. The water in the matrix depends on the volume and structure of the myofiber, whether raw or cooked, just like ST.

The differences in content and structure of IMCT may be attributed to the results of transversal shrinkage (Figure 4a). The stability of collagen fibers of LT was better than ST. The thermal transformation of collagen is gradual and greatly reliant on the ability of collagen fibers to contract [36]. As the heat proceeds, the strength of heat-insoluble collagen showed different trends in content. There are some differences in IMCT among muscles (Figure 4b), which are related to variations in the thermal shrinkage force in the perimysium among muscles [48]. There was a positive correlation between *T*_22_ and heat-soluble collagen content. The higher heat solubility of collagen correlated to the higher mobility of entrapped protons. Accordingly, that reflected a looser myofibrillar structure most probably because of the bigger intrinsic pores as a consequence of heat-induced shrinkage [49]. As was shown in Figure 4b, the samples with higher WHC showed a larger amount of total and heat-soluble collagen content, i.e., ST. The increase in collagen heat solubility indicated a decrease in the stability of the IMCT structure. The increasing content and thermal stability of collagen in the muscle were largely determined by its cross-links [50]. The presence of thermal-stable bonds means that intermolecular linkages are held at these temperatures. From the present study, it is inferred that the contraction of heat-insoluble IMCT on WHC may depend to some extent on the proteolysis and structural strength changes of IMCT components rather than solely caused by quantitative changes of IMCT.

## 4. Conclusions

To conclude, ST showed the best WHC during cooking compared to LT and SM. Under the same heating conditions, ST indicated lower WBSF, surface hydrophobicity and decorin, higher sarcomere length, collagen content and heat solubility. Apart from heat-induced myofiber shrinkage ratio, the volume of muscle fiber plays an important role in WHC. With larger myofiber volume, the muscle possesses much more space to retain water. The results of IMCT indicated that the amount and structural changes, such as heat-resistant cross-links may synergistically contribute to changes in WBSF. The quantity and quality of heat-induced IMCT were critical during the heat-induced shrinkage. The heat-soluble collagen content would influence the mobility of immobilized water. Further research is needed to understand the heat solubility changes of collagen during cooking.

## Figures and Tables

**Table 1:** WBSF and water holding capacity (means ± SE, n = 11) of porcine *longissimus thoracis*, *semimembranosus* and *semitendinosus*.

	LT	SM	ST
WBSF (N)	70.48 ± 3.95 ^a^	66.74 ± 1.86 ^a^	51.33 ± 2.73 ^b^
Cooking loss (%)	36.98 ± 0.77 ^a^	35.47 ± 0.90 ^a^	32.74 ± 0.96 ^b^
*T*_21_ (ms)	0.31 ± 0.00 ^a^	0.34 ± 0.02 ^a^	0.31 ± 0.02 ^a^
*T*_22_ (ms)	22.14 ± 0.53 ^c^	24.94 ± 0.48 ^b^	28.47 ± 0.55 ^a^
*T*_23_ (ms)	217.48 ± 9.53 ^b^	250.43 ± 15.20 ^ab^	271.46 ± 19.50 ^a^
*A*_21_ (%)	5.04 ± 0.00 ^a^	4.45 ± 0.00 ^b^	3.66 ± 0.00 ^c^
*A*_22_ (%)	91.48 ± 0.00 ^b^	93.00 ± 0.00 ^a^	93.01 ± 0.00 ^a^
*A*_23_ (%)	2.70 ± 0.00 ^ab^	2.28 ± 0.00 ^b^	3.30 ± 0.00 ^a^

Different superscript letters within a row indicate significant differences (*p* < 0.05). *T*_21,_ *T*_22_ and *T*_23_ represent the spin–spin relaxation time of bound water, immobile water and free water, respectively. *A*_21_, *A*_22_ and *A*_23_ represent the relative percentages of bound water, immobile water and free water, respectively.

**Table 2:** Volume shrinkage (means ± SE, n = 11) of porcine *longissimus thoracis*, *semimembranosus* and *semitendinosus*.

	LT	SM	ST
Volume shrinkage (%)	53.73 ± 1.62 ^a^	45.85 ± 2.31 ^b^	41.97 ± 2.40 ^b^
Transversal shrinkage (%)	37.75 ± 2.51 ^a^	33.09 ± 2.74 ^a^	31.81 ± 1.62 ^b^
Longitudinal shrinkage (%)	44.53 ± 1.57 ^a^	37.25 ± 1.84 ^b^	37.03 ± 2.77 ^b^
Sarcomere length—raw (μm)	1.29 ± 0.03 ^b^	1.37 ± 0.09 ^b^	1.63 ± 0.11 ^a^
Sarcomere length—cook (μm)	1.14 ± 0.03 ^b^	1.23 ± 0.06 ^b^	1.38 ± 0.05 ^a^
Fiber diameter—raw (μm)	70.57 ± 1.50 ^b^	79.05 ± 1.76 ^a^	82.52 ± 1.67 ^a^
Fiber diameter—cooked (μm)	63.17 ± 2.55 ^a^	70.24 ± 2.10 ^a^	70.97 ± 3.31 ^a^

Different superscript letters within a row indicate significant differences (*p* < 0.05).

**Table 3:** Protein denaturation characterization (means ± SE, n = 11) of porcine *longissimus thoracis*, *semimembranosus* and *semitendinosus*.

	LT	SM	ST
Bound BPB content (μg)	181.15 ± 5.49 ^a^	156.50 ± 7.02 ^b^	128.64 ± 10.28 ^c^
Total collagen (mg/g DM)	13.85 ± 1.98 ^b^	17.94 ± 1.15 ^ab^	18.87 ± 1.33 ^a^
Heat Soluble collagen (mg/g DM)	3.50 ± 0.49 ^c^	4.96 ± 0.42 ^b^	7.12 ± 0.45 ^a^
Cross-links (μg/g collagen)	2.93 ± 0.82 ^a^	1.54 ± 0.24 ^ab^	1.34 ± 0.21 ^b^
Decorin (μg/g collagen)	15.27 ± 2.18 ^a^	11.80 ± 1.31 ^a^	10.80 ± 1.06 ^a^

Different superscript letters within a row indicate significant differences (*p* < 0.05).

## Data Availability

Data are contained within the article.
